# DegP Chaperone Suppresses Toxic Inner Membrane Translocation Intermediates

**DOI:** 10.1371/journal.pone.0162922

**Published:** 2016-09-14

**Authors:** Esther Braselmann, Julie L. Chaney, Matthew M. Champion, Patricia L. Clark

**Affiliations:** 1 Department of Chemistry & Biochemistry, University of Notre Dame, Notre Dame, Indiana, United States of America; 2 Department of Chemical & Biomolecular Engineering, University of Notre Dame, Notre Dame, Indiana, United States of America; Centre National de la Recherche Scientifique, Aix-Marseille Université, FRANCE

## Abstract

The periplasm of Gram-negative bacteria includes a variety of molecular chaperones that shepherd the folding and targeting of secreted proteins. A central player of this quality control network is DegP, a protease also suggested to have a chaperone function. We serendipitously discovered that production of the *Bordetella pertussis* autotransporter virulence protein pertactin is lethal in *Escherichia coli* Δ*degP* strains. We investigated specific contributions of DegP to secretion of pertactin as a model system to test the functions of DegP *in vivo*. The DegP chaperone activity was sufficient to restore growth during pertactin production. This chaperone dependency could be relieved by changing the pertactin signal sequence: an *E*. *coli* signal sequence leading to co-translational inner membrane (IM) translocation was sufficient to suppress lethality in the absence of DegP, whereas an *E*. *coli* post-translational signal sequence was sufficient to recapitulate the lethal phenotype. These results identify a novel connection between the DegP chaperone and the mechanism used to translocate a protein across the IM. Lethality coincided with loss of periplasmic proteins, soluble σ^E^, and proteins regulated by this essential stress response. These results suggest post-translational IM translocation can lead to the formation of toxic periplasmic folding intermediates, which DegP can suppress.

## Introduction

Although all bacterial proteins are synthesized in the cytoplasm, many are subsequently secreted to the cell surface, where they function to sense and respond to the cellular environment. In *Escherichia coli*, approximately 10% of proteins contain an N-terminal signal sequence for translocation across the inner membrane (IM) to the periplasm [1]. Most bacterial secreted proteins, including those used for virulence, enter the periplasm through the primary export channel SecYEG [2–4], either co- or post-translationally. While the rate of co-translational translocation is limited by the rate of protein synthesis [3,4], post-translational translocation can occur ~10-fold faster [5,6]. Most secreted proteins in *E*. *coli* are thought to be translocated post-translationally [2,7]. The effects of changing the IM translation mechanism on downstream steps in the periplasm are poorly understood, but there is some evidence that the IM translocation mechanism can affect the conformation of the secreted protein after its translocation [5,8,9], which could lead to misfolding and cell envelope stress.

In the periplasm, secreted proteins encounter several abundant chaperones that comprise an efficient quality control network [10–12]. Periplasmic chaperones often have overlapping substrate specificities [13], which has made it challenging to dissect contributions of individual chaperones to specific aspects of protein quality control. For example, the periplasmic chaperone DegP can function as either a chaperone or a protease [14–16]. The main substrates of the DegP protease are OM β-barrel proteins (OMPs) [17], but it has been more difficult to identify its contributions as a chaperone. As a result, most of our current understanding of the DegP chaperone activity is from *in vitro* studies [18–20]. During heat-shock, *E*. *coli* Δ*degP* is lethal, but growth can be rescued by production of a chaperone-only DegP mutant, suggesting that DegP may function as a chaperone for OMPs during their assembly in the OM [21,22]. However, recent studies have shown that while assembly-defective OMPs are sequestered by DegP during heat shock, DegP does not promote OM assembly of OMPs and functions primarily as a protease in *E*. *coli* [17].

**Fig 1:**
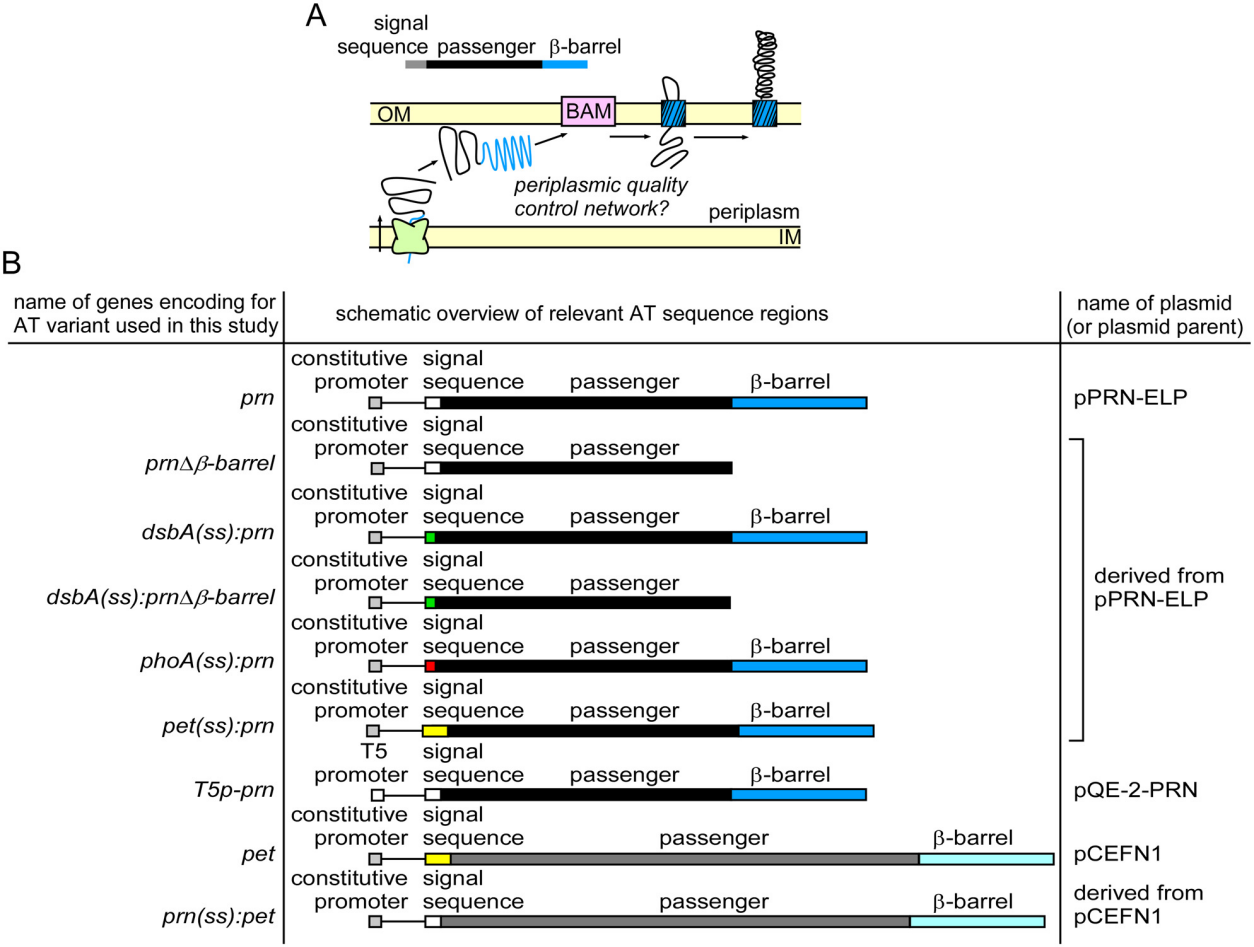
The autotransporter (AT) secretion pathway. (A) Schematic overview of AT secretion mechanism. The passenger domain remains in a non-native, unfolded conformation during transit across the periplasm. (B) Constructs used in this study. The lengths of the AT variants are drawn to scale and include annotations of promoters and regions encoding annotated domains.

We serendipitously discovered that DegP is required during production of a *B*. *pertussis* autotransporter (AT) virulence protein in *E*. *coli*. AT proteins are the largest family of virulence proteins secreted from Gram-negative pathogens, including *E*. *coli* [23]. The AT secretion mechanism consists of three discrete steps (Fig 1A): (*i*) The N-terminal AT signal sequence mediates translocation of the AT precursor across the IM, (*ii*) the C-terminal β-barrel domain folds and inserts into the outer membrane (OM), where it assists with (*iii*) translocation of the central AT passenger across the OM to the cell surface, assisted by the β-barrel assembly machinery (BAM) and the translocation and assembly module (TAM) [24–31]. After OM translocation, the AT passenger is typically cleaved from its β-barrel [32]. Much work has been devoted to understanding the OM translocation portion of AT secretion, but details of earlier steps, including IM translocation, are less well understood. We and others have shown that the AT passenger remains in an unstable, non-native conformation while it resides in the periplasm [33–36]. This unstable, non-native conformation is required for the passenger to remain compatible with OM translocation [24,29,37,38]. ATs therefore represent unique model substrates to dissect how the *E*. *coli* periplasmic chaperone network handles the accumulation of unstable, unfolded substrates during AT secretion.

We found that the DegP chaperone activity is required during IM translocation of the *B*. *pertussis* pertactin AT passenger in *E*. *coli*. We used this model system to investigate the basis for DegP dependent growth and found that changing the AT IM translocation mechanism from post- to co-translational was sufficient to relieve the DegP chaperone requirement. An analysis of the proteome revealed that failure of the σ^E^ periplasmic stress response was sufficient to explain lethality. These results provide unique insights into the periplasmic protein quality control network and bacterial cell death triggered by early steps in secretion of an extracellular protein.

## Materials and Methods

### Construction of plasmids encoding autotransporter (AT) variants and summary of *E*. *coli* strains

The genotypes of the AT variants used in this study and the plasmids used to express these genes are summarized in Fig 1B. A plasmid to express the pertactin passenger alone was described previously as pPERPLC01; the protein product accumulates as inclusion bodies in the *E*. *coli* cytosol [39]. Plasmid pCEFN1 was used to express *pet* under the control of its endogenous promoter [40]. All plasmids were constructed using standard molecular biology techniques. All primers were purchased from IDT. *E*. *coli* DH5α (Invitrogen) was used for cloning procedures.

Plasmid pPRN-ELP, to express *prn* from a constitutive *tac* promoter, was generated from its parents pP.93WT [29] and pPERPLC01 [39]. Three point mutations present in pP.93WT were removed by ligating a portion of the gene encoding the passenger region from pPERPLC01 into pP.93WT. To do this, both plasmids were digested using at the unique *Kpn*I and *Rsr*II sites and the encompassing region from pPERPLC01 with the correct sequence was ligated in pP.93WT. As a result, *prn* differs from the gene encoded by pP.93WT in three positions (resulting in three amino acid substitutions: D262E, P264L, S332P). All *prn* gene variants used in this study were in this background (D262E, P264L, S332P).

Construct *T5p-prn* was made by cloning the *prn* sequence in pQE-2 (Qiagen), resulting in plasmid pQE-2-PRN. First, a *BamH*I site was introduced in pQE-2 by site-directed mutagenesis using primers pQE2 BamHI SDM FW and pQE2 BamHI SDM RV (S1 Fig), resulting in the plasmid pQE-2-BamHI. The *BamH*I site was located downstream of the region encoding an N-terminal His-tag in pQE-2 to ensure that the pertactin gene would not have a His-tag. The coding sequence of pertactin was amplified from *prn* using primers PCR amp P.93 BamHI FW and PCR amp P.93 HindIII RV (S1 Fig) and ligated in pQE-2-BamHI via the *BamH*I and *Hind*III sites, resulting in plasmid pQE-2-PRN.

Signal sequences of ATs were exchanged using MEGAWHOP PCR [41] and the primers listed in S1 Fig. A pertactin deletion variant corresponding to residues 631–910 lacked the region coding for the C-terminal β-barrel domain of pertactin. This mutant was generated by introducing a stop codon in the corresponding parent plasmid using primers N631Stop FW and N631Stop RV (S1 Fig).

*E*. *coli* KS303 (F- Δ*lacX74*, *galE*, *galK*, *phoA* Δ*PvuII*, *StrR lpp*) and KS476 (KS303 *degP41*::*km*) are derivatives of MC1000, described previously [38,42]. Both strains were cultured at 30°C. The *E*. *coli* chaperone deletion strain series are derivatives of BW30270, described previously [43]. The following deletion strains from this series were used for colony forming unit (CFU) measurements as described below: JB4 (*fkbA*::*cm*), JB117 (*skp*::*kan*), and JB8 (*degP*::*kan*).

### Growth curves

All cultures were grown at 30°C. To compare growth of different strains in parallel, cultures were first normalized during inoculation from overnight cultures as follows: The overnight culture with the highest optical density (OD_600_) was inoculated at a 1:50 dilution and the remaining cultures were normalized to have the same starting OD_600_. After growth to log phase (~OD_600_ = 0.4), fresh cultures were once again started to ensure that all cultures started at a similar growth phase. For the second inoculation, the equivalent of a 1:50 dilution from a culture corresponding to OD_600_ = 2.5 was used. The growth curve after this second inoculation was recorded.

### Cell viability assay

To quantify the phenotypic effects of ATs on *E*. *coli* growth in different genetic backgrounds, the viability of *E*. *coli* (KS303 and KS476) was quantified after plasmid transformations. In each transformation reaction, 1 μL of plasmid DNA from a 50 ng μL^-1^ stock was added to 50 μL of chemically competent *E*. *coli*. In some cases, a plasmid encoding a variant of *degP* (*degP S210A*) expressed from a *trc* promoter (pCS10) [16] was co-transformed. The gene product from this *degP* variant is a protease deficient version of DegP. After heat shock transformation for 45 s at 42°C, 200 μL of SOC medium (2% tryptone, 0.5% yeast extract, 10 mM NaCl, 2.5 mM KCl, 10 mM MgCl_2_, 10 mM MgSO_4_, 20 mM glucose) was added and cells were allowed to recover for 1 h at 30°C. Transformation reactions were diluted in SOC medium and plated on LB-agar supplemented with appropriate antibiotics (100 μg mL^-1^ ampicillin (Amp) for single transformations with plasmids encoding AT variants, plus 100 μg mL^-1^ and 35 μg mL^-1^ chloramphenicol (Cam) for co-transformations with pCS10).

### Mass spectrometry

*E*. *coli* KS303 or *E*. *coli* KS476 transformed with pQE-2-PRN (Fig 1B) were grown at 30°C. For each sample, 200 mL LB medium supplemented with 100 μg mL^-1^ Amp was inoculated with an overnight culture at a 1:50 dilution. Expression of *prn* was induced with 0.5 mM IPTG at the indicated OD_600_, and cells were pelleted at the indicated times. Cell pellets were lysed using a bead beater (BioSpec) in 50 mM Tris pH 8.0, 1% TritonX-100, 150 mM KCl, 1mM EDTA, 1mM PMSF, and clarified by centrifugation at 12,000 x *g* for 12 min.

Total protein were quantified using Micro BCA (Pierce) according to the manufacturer’s instructions, and 100 μg of each lysate was precipitated and digested with trypsin using the FASP protocol as described previously [44–46]. All reagents were purchased from Sigma unless otherwise indicated. Briefly, Tris(2-carboxyethyl)phosphine-reduced and iodoacetamide alkylated samples were buffer-exchanged using filtration concentrators (Amicon; 10,000 MW cutoff) with five washes of 8 M urea in 100 mM Tris pH 8.0, then exchanged with two washes of 50 mM ammonium bicarbonate pH 8.0. One μg of trypsin (Promega) was added to each sample and digested at 37°C for 8 h. Peptides were recovered from the filtration concentrator by spinning and a second spin with addition of 100 μl of 500 mM NaCl and quenched with addition of 5% trifluoroacetic acid. Peptide mixtures were desalted using C18 SPE spin columns (Protea Sciences), dried and resuspended in 0.2% formic acid for LC/MS/MS analysis.

LC/MS/MS was performed as described previously [44]. Briefly, 750 ng of each sample was analyzed in duplicate and separated over a 90 min gradient using 3–40% water, 0.1% formic acid—acetonitrile, 0.1% formic acid (Honeywell Burdick & Jackson) using a 100 μm x 100 mm C18BEH column (Waters) running at 900 nL min^-1^. MS/MS was performed on an LTQ Velos Orbitrap mass spectrometer running a TOP12 method.

### Proteomic data analysis

MS/MS files were searched using the Andromeda search engine [47] against the current *E*. *coli* FASTA database supplemented with laboratory contaminants and the sequence of pertactin (*B*. *pertussis*) produced in these strains. Deamidation of NQ, oxidation of M, carboxyamidomethyl-C, and N-terminal acetylation were considered as modifications. The peptide-spectral matches false-discovery rate (FDR) was set to 0.01%. Protein FDR was set to 0.02% using target-decoy false discovery calculation [48].

Protein quantification was performed using label-free-quantification based on extracted ion chromatograms. MaxLFQ within MaxQuant v 1.5.2.8 software was used for peak integration and normalization [49]. Suggested settings were used for integration. Two or greater quantified peptides per protein were required and normalization against total protein and corrected mass spectrometry response was performed. Deletion of DegP was readily verified within these quantifications (panel A in S2 Fig). Quantitative data is given as the log2 transformed fold-change *versus* the *E*. *coli* KS303 strain after 2 h of pertactin induction. Error bars are plotted as standard error (∑/mean) for each label-free measurement. RAW data files, search results, search parameters and databases were deposited into MassIVE and are available for download at massive.ucsd.edu with the identifier MSV000080095 or ftp://MSV000080095@massive.ucsd.edu.

To estimate abundance of periplasmic proteins, the 1000 most abundant proteins across all conditions were analyzed further. Out of this set, 39 proteins have ‘periplasm’ in their name. These proteins were used as a proxy for all periplasmic proteins. The quantity of these periplasmic proteins during death (5 h post induction, KS476) was compared to the control condition (2 h time point, KS303, no induction). Out of all 39 periplasmic proteins identified in this way, six did not change significantly or increased and one had ambiguous quantification. The remaining 32 proteins (82% of total) decreased by at least two-fold during death.

### Measuring accumulation and secretion of AT variants

Accumulation and secretion of AT variants *in vivo* was verified by measuring the appearance and proteolytic processing of the AT precursor in cell lysates using western blotting, as described previously [24,25,29,32,50]. *E*. *coli* were transformed with plasmids encoding the indicated genes and grown overnight. The equivalent of 1 mL of cells at OD_600_ = 0.5 were pelleted for 5 min at 8,000 x *g* and resuspended in 50 μL SDS loading buffer. For cells where variants of the Pet passenger were produced, a sample of the growth medium was collected to quantify the secreted passenger. After removing bacteria by centrifugation, the spent growth medium was filtered through a 0.22 μm filter and diluted 15-fold with SDS loading buffer. WCL and spent media samples were boiled for 5 min, separated by SDS-PAGE and analyzed by western blotting as described below.

### Western blotting

Samples for western blot analysis were separated by SDS-PAGE and transferred to a polyvinylidene fluoride (PVDF) (Bio-Rad) membrane as follows. The membrane was wet in methanol, and then both the membrane and the SDS-PAGE gel were equilibrated in western transfer buffer (WTB, 25 mM Tris base, 192 mM glycine, 20% (v/v) methanol) for 10–15 min. The gel was transferred to the PVDF membrane at 4°C for 2 h at 70 V. After transfer, the membrane was equilibrated for 5 min in 20 mL Tris buffered saline Tween-20 (TBST) consisting of 20 mM Tris HCl pH 7.5, 150 mM NaCl and 0.5% (v/v) Tween-20 supplemented with 5% (w/v) powdered dry milk (PDM). The primary antibody was added to the TBST/5% PDM solution and incubated while shaking. The membrane was washed with 20 mL TBST three times to remove unbound antibody followed by incubation with the secondary antibody in 20 mL TBST/5% PDM. Then, the membrane was incubated for 30 min with an alkaline phosphatase (AP)-conjugated secondary antibody and washed three times with 20 mL TBST to remove unbound antibody. The membrane was rinsed with ddH_2_O five times and developed by incubation with 10 mL alkaline phosphatase (AP) buffer (100 mM Tris HCl, pH 9.5, 100 mM NaCl, 5 mM MgCl_2_) containing 66 μL of nitro blue tetrazolium and 33 μL of 5-bromo-4-chloro-3-indoyl-phosphate (Promega). The blot was rinsed with water to stop the reaction.

The primary antibodies were a polyclonal rabbit anti-pertactin antibody (1:7,000 dilution) [29], a polyclonal rabbit anti-Pet antibody (1:7,000 dilution) [40] or a polyclonal rabbit anti-MBP/DegP antibody (1:15,000 dilution) [51]. The secondary antibody was an AP-coupled goat anti-rabbit IgG antibody (Novus Biologicals, Littleton, CO, 1:10,000 dilution).

## Results

### Constitutive expression of *prn*, but not *pet*, is lethal in the absence of DegP

**Fig 2:**
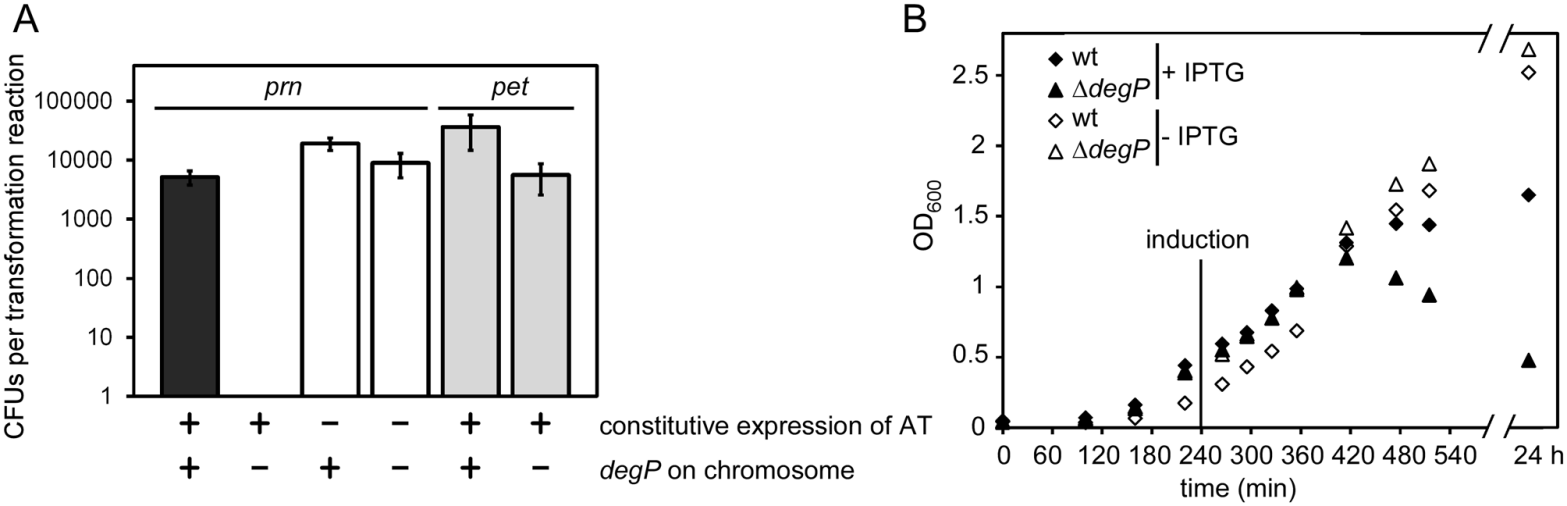
Expression of *prn* in an *E*. *coli degP* deletion strain is lethal. (A) Viability of the *E*. *coli degP* null strain versus its parent strain was assessed after transforming plasmids that constitutively express AT variants. Viability was measured by counting colony forming units (CFUs); see Methods. Error bars are standard deviations from three biological repetitions. (B) Growth of the *E*. *coli degP* null strain versus its parent strain, with or without IPTG-induction of *prn* expression. Shown are representative growth curves.

To systematically investigate the effects of deleting *degP* during AT expression, we compared *E*. *coli* growth during the synthesis of two dissimilar ATs, *Bordetella pertussis* pertactin (gene name: *prn*) [24,29,39] and Pet, an AT from the *s*erine *p*rotease *a*uto*t*ransporters of *E*nterobacteriaceae (SPATE) subfamily [24,40,52] (Fig 1B). Surprisingly, expression of *prn* in the absence of DegP was lethal: transformation of a *degP* null strain with a plasmid to constitutively express *prn* yielded no colonies (Fig 2A). Likewise, induction of *prn* expression in the *degP* deletion background resulted in a dramatic decrease in cell density (OD_600_; Fig 2B), a characteristic phenotype of cell death. Such conditional synthetic lethality has not previously been observed during AT expression in the absence of DegP. Past studies have indicated DegP deletion has no significant effect on *E*. *coli* growth, with or without AT expression [38]. Consistent with these studies, we observed that transforming a plasmid to constitutively express *pet* did not affect viability of *E*. *coli* in the absence of DegP (Fig 2A).

### The DegP chaperone function is required for *E*. *coli* survival during pertactin production and secretion

**Fig 3:**
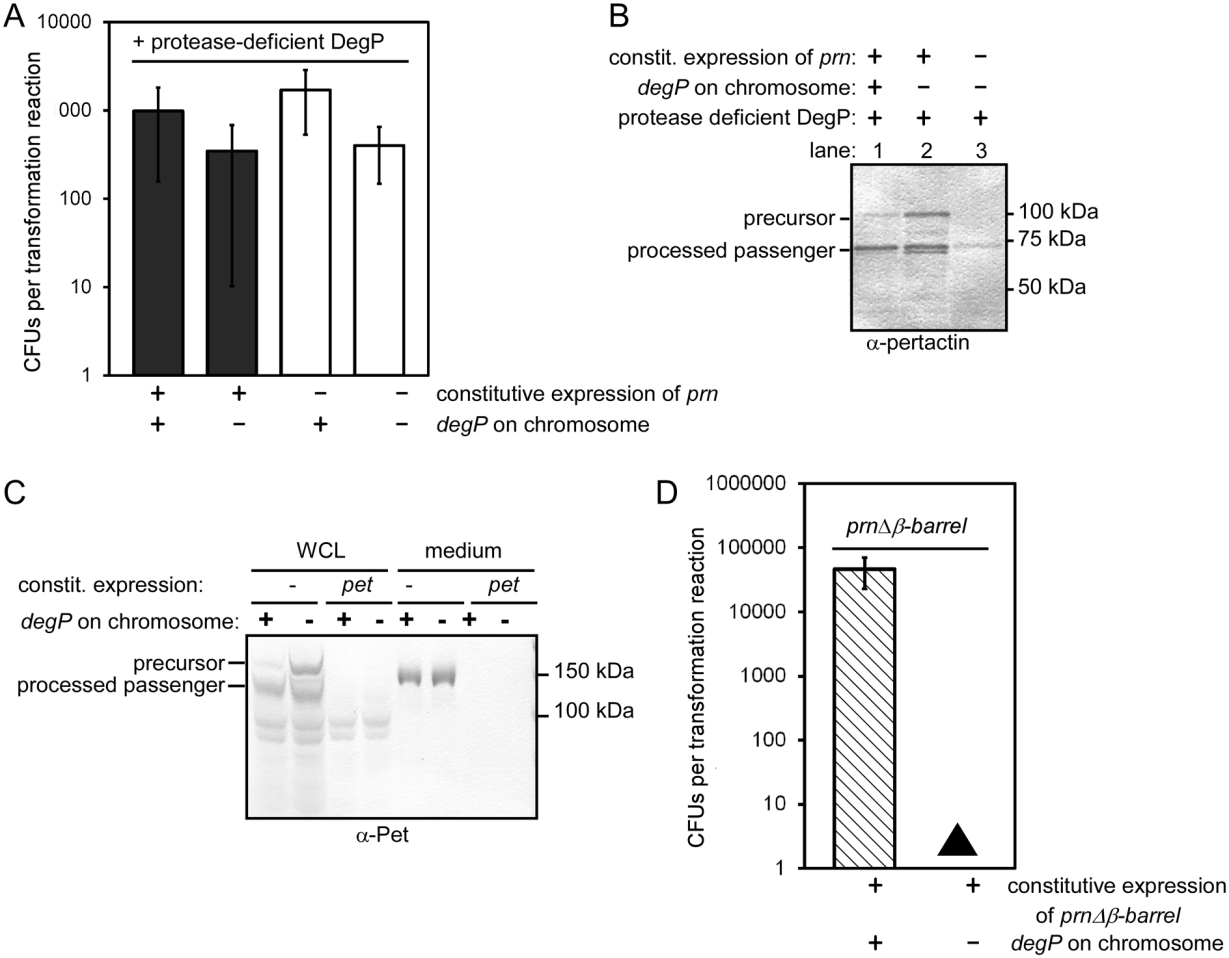
Differential requirement of the DegP protease and chaperone activity during expression of model ATs. (A) *E*. *coli* viability as a function of *degP*, with or without constitutive expression of *prn*. To test if the DegP chaperone alone is sufficient to restore viability, a protease deficient version of DegP [16] was produced by co-transforming a plasmid expressing *degP S210A*. (B) Expression of *prn* results in production of the precursor and processing of the passenger (samples shown here were collected from cells described in (A). The role of the DegP protease for pertactin accumulation and secretion was assessed by expressing *prn* when only the DegP chaperone was present, versus the DegP chaperone plus the chromosomal wild type DegP. In the presence of the DegP protease activity, less pertactin precursor accumulates (compare lanes 1 vs. 2). (C) Wild type *pet* was expressed overnight in *E*. *coli* with or without a chromosomal copy of *degP* (same samples as grey bars in Fig 2A). In the absence of DegP, more full-length precursor accumulates in whole cell lysates (WCL). The yield of secreted Pet in the media after overnight production was not significantly altered with or without DegP. (D) After transforming *prn*Δ*β-barrel* in the *E*. *coli degP* null strain, very small colonies appeared after prolonged incubation (black triangle). (A, D) Error bars are standard deviations from at least three biological repetitions.

To dissect the specific contributions of the chaperone *vs*. protease function of DegP to the observed lethality, we first determined whether the DegP chaperone activity alone was sufficient to rescue the lethal phenotype. We transformed a plasmid to constitutively express *prn* in the *E*. *coli degP* deletion strain as before, but also co-transformed a plasmid encoding a protease-deficient version of DegP that retains the DegP chaperone activity (DegP S210A) [16]. Supplying the DegP chaperone function was sufficient to restore *E*. *coli* growth during *prn* expression (Fig 3A).

We next asked whether supplying the DegP chaperone rescues pertactin secretion, in addition to enabling *E*. *coli* growth. We and others have previously shown that pertactin processing is a reliable assay for secretion across the OM; secretion-impaired mutants of ATs remain unprocessed in the periplasm [24,25,28,29,53,54]. We observed that the processed pertactin passenger accumulated to similar levels when the DegP chaperone variant was produced (Fig 3B), demonstrating that the DegP chaperone is sufficient to restore growth and allow pertactin secretion in *E*. *coli*.

Next, we tested the role of the DegP protease during AT secretion in our model systems. We asked whether the pertactin precursor is also a substrate for the DegP protease while critically requiring the DegP chaperone. We constitutively expressed *prn* in *E*. *coli* strains with or without *degP* on the chromosome. To avoid the lethal phenotype described above, we also supplied the DegP chaperone activity by producing the protease deficient DegP variant from a plasmid in both strains (Fig 3B). We observed that the processed pertactin passenger accumulated to similar levels with or without the DegP protease activity (Fig 3B), indicating that the DegP protease activity was not crucial for pertactin secretion or cell viability. However, the pertactin precursor accumulated to higher levels when the protease activity of DegP was omitted (Fig 3B, compare lanes 1 and 2), indicating that DegP degraded a portion of the pertactin precursor pool. We tested the DegP protease activity during expression of *pet* as well (Fig 3C). As seen for pertactin, absence of the DegP protease resulted in an increase in the Pet precursor pool, but no detectable changes for passenger secretion efficiency. Taken together, these results demonstrate that DegP, when present, degrades a portion of both the pertactin and Pet precursors. This result is consistent with previous reports of DegP degradation of AT secretion intermediates in the periplasm [38,54,55].

We next tested whether *prn* expression was dependent on other *E*. *coli* chaperones besides DegP. To do this, we turned to an existing series of *E*. *coli* chaperone deletion strains [43]. We transformed the *degP* deletion strain from this series (JB8) with a plasmid constitutively expressing *prn* and confirmed that, as for the *degP* deletion strain KS476, transformation with the *prn* plasmid results in lethality (panel A in S3 Fig). In contrast, strains lacking FkpA (JB4; *fkpA*::*cm*) or Skp (JB117; *skp*::*kan*) were viable after transformation (panel A in S3 Fig) and led to efficient secretion of pertactin (panel B in S3 Fig). These results demonstrate that despite the known overlapping substrate specificity amongst periplasmic chaperones [13], lethality upon *prn* expression in *E*. *coli* is related to a specific requirement for the DegP chaperone activity.

### Lethality in the absence of DegP occurs prior to pertactin OM translocation

Previous reports suggested that DegP contributes a quality control function during AT OM translocation [37,56,57]. To test whether the lethality of *prn* expression in the absence of DegP is related to pertactin OM translocation, versus an earlier step in secretion, we constructed *prn*Δ*β-barrel* (Fig 1B). Because the product of this construct lacks the C-terminal OM β-barrel, it will neither integrate into the OM nor will the passenger translocate across the OM [58]. If lethality was related to pertactin β-barrel OM assembly or OM translocation, deleting this domain should reverse the lethal phenotype. However, expression of *prn*Δ*β-barrel* in the *degP* deletion strain resulted in a severely toxic phenotype (Fig 3D), yielding only extremely small colonies after prolonged incubation (see Table 1 for a comparison of relevant phenotypes). The severe growth defect of *prn*Δ*β-barrel* in the absence of DegP indicates that DegP plays a crucial role in early steps of pertactin secretion, prior to OM translocation.

**Table 1:** Summary of phenotypes observed upon AT expression in the *E*. *coli degP* deletion strain.

	*prn*	*phoA(ss)*:*prn*	*prn*Δ*β-barrel*	*pet(ss)*:*prn*	*prn(ss)*:*pet*	*dsbA(ss)*:*prn*Δ*β*-*barrel*	*dsbA(ss)*:*prn*	*pet*
Cell growth	**-**	**-**	**+**	**++**	**+++**	**+++**	**+++**	**+++**
Precursor accumulation	N/A	N/A	**++**	**++**	**+**	**+++**	**+++**	**++++**
Passenger secretion	N/A	N/A	N/A	**++**	**+**	N/A	**+++**	**+++**

A phenotype similar to expression in the wild type *E*. *coli* parent strain was scored as (+++), whereas absence of the phenotype in comparison to the parent strain was scored as (-). Note that precursor accumulation of wild type Pet is increased in the *degP* deletion strain versus the parent (++++). See Fig 1B for details on these constructs.

### Fusion of pertactin to a co-translational signal sequence is sufficient to suppress DegP chaperone dependency

**Fig 4:**
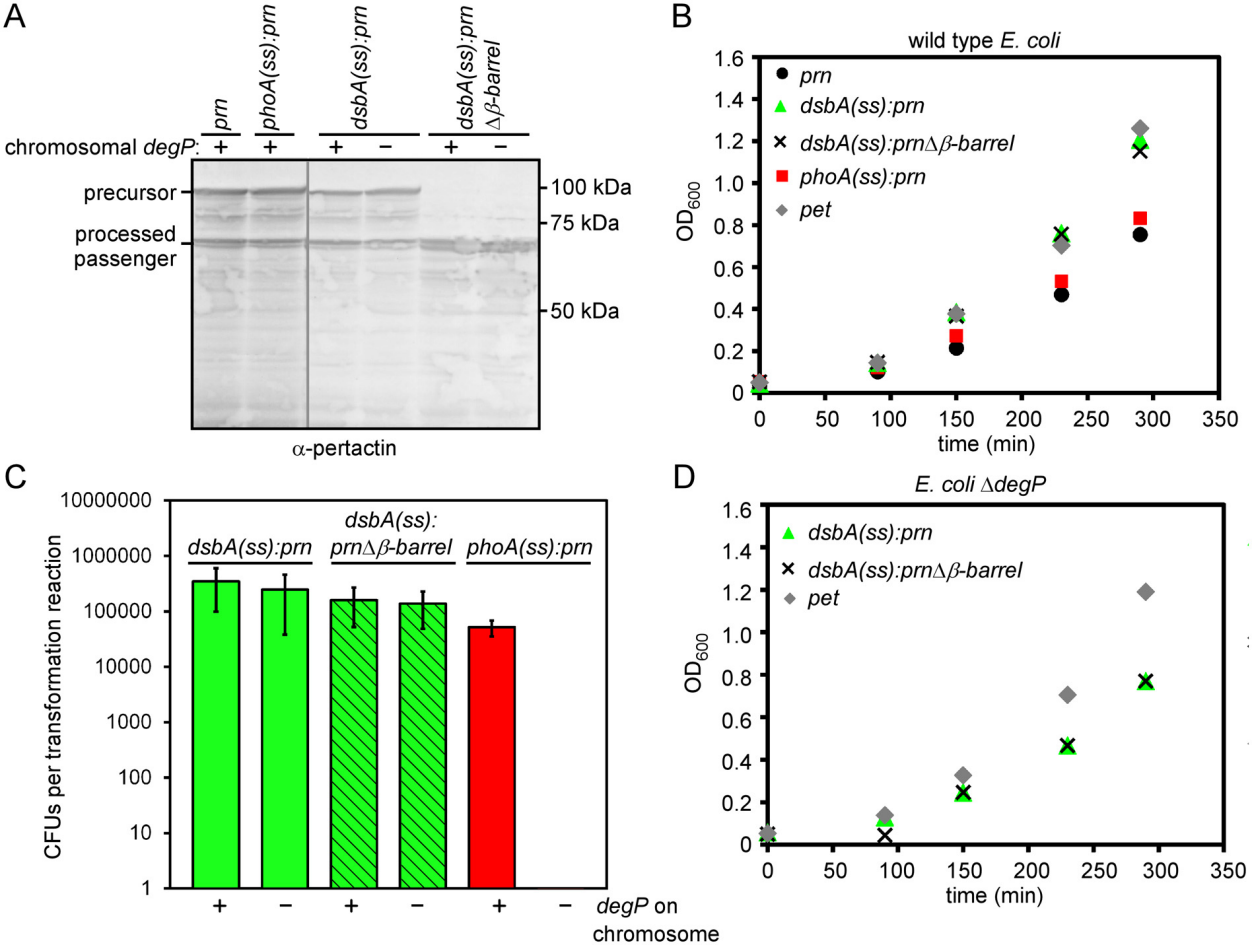
The co-translational DsbA signal sequence, but not the post-translational PhoA signal sequence, bypasses lethality. (A) Production and secretion of pertactin chimera constructs in the *E*. *coli degP* null strain versus its parent. Bacteria were grown to stationary phase and protein production was analyzed by western blotting of whole cell lysates. Successful secretion of the pertactin passenger is measured by appearance of the processed passenger [24,25,29,32,50]. Two non-adjacent portions of the same blot are shown (grey line). (B) Expression of signal sequence chimera constructs does not dramatically affect growth of wild type *E*. *coli*. (C) Plasmids to express *dsbA(ss)*:*prn* and *phoA(ss)*:*prn* under control of a constitutive promoter were transformed in the *E*. *coli degP* null mutant versus its parent as described in Fig 2A. Error bars are standard deviations from at least three biological replicates. (D) Expression of *dsbA(ss)*:*prn*, *dsbA(ss)*:*prn*Δ*β-barrel* and *pet* does not affect growth in the *degP* null strain. (B, D) Shown are representative growth curves.

We next sought to identify what features of pertactin, but not Pet, trigger lethality in the *degP* deletion background during early secretion steps. A notable difference lies within the signal sequences: the Pet signal sequence has an N-terminal extension specific to the SPATE sub-family of AT proteins [59,60]. Although the precise function of this N-terminal signal sequence extension remains unclear, it has been proposed to slow down the rate of IM translocation [60–65]. To systematically test the effects of altering the IM translocation mechanism on DegP-dependent *prn* expression, we replaced the pertactin signal sequence with a well-characterized signal sequence from *dsbA* or *phoA*, to generate *dsbA(ss)*:*prn* and *phoA(ss)*:*prn* (Fig 1B). DsbA is known to be translocated co-translationally across the IM [66], whereas PhoA IM translocation is mainly post-translational [5,67]. Both of these *E*. *coli* signal sequences are routinely used to target proteins to the periplasm [67–69]. As expected, neither of these signal sequences altered pertactin production or secretion in the parent *E*. *coli* strain (Fig 4A). Likewise, we observed no significant changes in growth rates (Fig 4B). However, we found that replacing the pertactin signal sequence with the co-translational signal sequence of *dsbA* was sufficient to bypass the lethality of expressing *prn* in the *degP* deletion strain and restore pertactin secretion (Fig 4). In contrast, fusion to the PhoA signal sequence was lethal (Fig 4C). The lethality of *phoA(ss)*:*prn* in in the *degP* deletion background indicates that post-translational translocation of pertactin across the IM is sufficient to recapitulate the conditional synthetic lethality observed for pertactin in the absence of DegP. Importantly, both *dsbA* and *phoA* are endogenous to *E*. *coli*. Recapitulation of both the lethal and viable phenotypes when these signal sequences were fused to pertactin confirms that the phenotype is directly linked to co- vs. post-translational IM translocation in *E*. *coli*.

### The extended Pet signal sequence can partially rescue lethality of pertactin in the absence of *degP*

The *dsbA(ss)*:*prn* and *phoA(ss)*:*prn* results indicate that the nature of the signal sequence or the resulting differences in IM translocation mechanism largely determined the DegP dependence, leading us to closely examine the differences in the Pet and pertactin signal sequences. Our current understanding of AT IM targeting is based on studies of the SPATE sub-family of AT proteins, all of which share the N-terminal signal sequence extension described above [59,60]. A survey of the literature revealed that DegP is dispensable during production of ATs with extended signal sequences, including IcsA [56,57,70], Hbp [34,38,54,55,64], EspP, Pet and other SPATEs [37], as well as the two-partner secretion (TPS) protein FHA [71,72]. Furthermore, the SPATE signal sequence extension appears to reduce the rate of post-translational IM translocation and/or release of the AT from the IM [60–65]. We hypothesized that, like the co-translational DsbA signal sequence, the extended SPATE signal sequence could similarly reduce DegP dependence.

**Fig 5:**
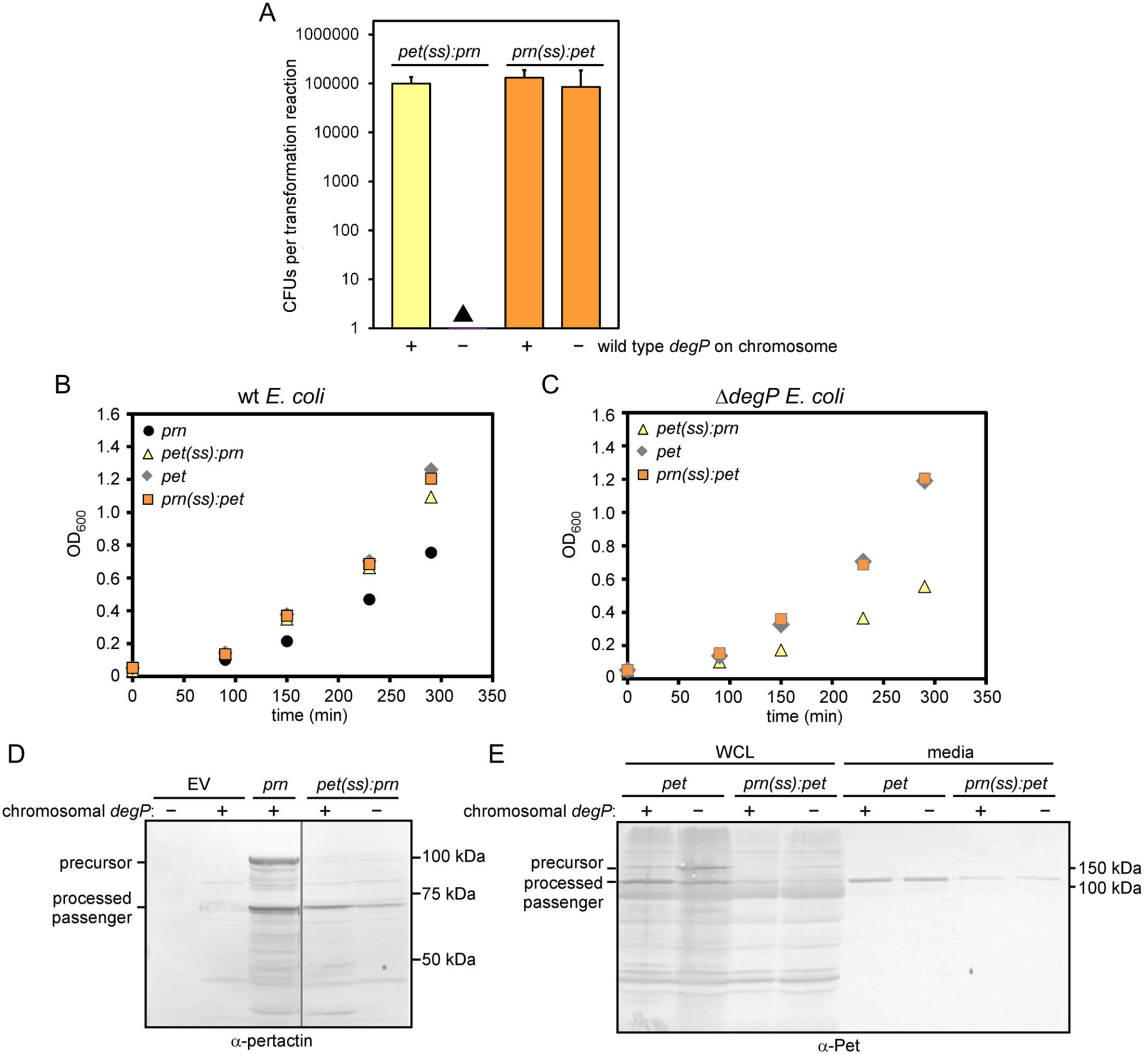
The Pet signal sequence partially rescues lethality of pertactin in the *degP* null strain. (A) The *degP* null strain and its parent were transformed with plasmids to express *pet(ss)*:*prn* or *prn(ss)*:*pet* as described in Fig 2A. Error bars are standard deviations from at least three biological replicates. *Filled triangle*: After transforming a plasmid to express *pet(ss)*:*prn* in the *degP* deletion strain, very small colonies appeared after prolonged incubation. (B) Growth of *E*. *coli* with a chromosomal copy of *degP* is not significantly altered when AT signal sequence chimera variants are produced. (C) Expression of *pet* and *prn(ss)*:*pet* does not affect growth in the *degP* deletion background, whereas expression of *pet(ss)*:*prn* reduces the growth rate. (D) The chimera *pet(ss)*:*prn* was expressed and the chimera protein was secreted in *E*. *coli* regardless of the presence or absence of DegP, but the secretion yield was reduced in the *degP* deletion strain. Two non-adjacent portions of the same blot are shown (grey line). (E) Expression of *prn(ss)*:*pet* resulted in accumulation of the chimera protein at reduced levels in *E*. *coli*. Likewise, secretion of the Pet passenger to the medium was reduced, regardless of the presence or absence of DegP. (B, C) Shown are representative growth curves.

To test whether the extended signal sequence from Pet can rescue lethality in pertactin, we replaced the region of *prn* encoding the signal sequence with the region encoding the Pet signal sequence (resulting in construct *pet(ss)*:*prn*; Fig 1B) and transformed the plasmid expressing this chimera into the *degP* deletion strain to assess viability. We observed small colonies after prolonged incubation, but these did not appear within the timeframe required to qualify as CFUs in our assay (Fig 5A). Note that, in contrast, we did not detect any colonies, even very small ones, when *prn* was transformed into the *degP* deletion strain (Fig 2A). We hypothesized that the colonies resulting from transformations with the plasmid harboring *pet(ss)*:*prn* might be viable, albeit with a strong growth defect. We tested growth of these small colonies in liquid culture. As a control, we transformed the *E*. *coli* parent strain with the plasmid to express *pet(ss)*:*prn* and no growth defect was observed in the *E*. *coli* parent (Fig 5B). As hypothesized, we were able to grow the *degP* deletion strain transformed with a plasmid to express *pet(ss)*:*prn* in culture (Fig 5C), albeit at a slow growth rate (see also Table 1). These results suggest that fusing the Pet signal sequence to pertactin improves viability of the *degP* deletion strain, albeit to a limited extent. To test directly if pertactin secretion was also rescued in this chimera context, we measured pertactin accumulation and secretion as described above. Indeed, mature pertactin accumulated in the *degP* deletion strain, although the secretion yield for the chimera was reduced (Fig 5D). The overall slower growth rate (Fig 5C) likely contributes to this reduced secretion yield. Taken together, these results indicate that the extended Pet signal sequence can partially rescue the lethal phenotype of pertactin in *E*. *coli*, although significant toxicity remains.

We next tested the reverse hypothesis: whether fusion of the pertactin signal sequence to Pet was sufficient to induce toxicity in the *degP* null strain. We transformed a plasmid encoding *prn(ss)*:*pet* (Fig 1B) in the *degP* deletion strain and observed cell growth similar to cells transformed with wild type *pet* (Fig 5A and 5C). In contrast to wild type Pet, there was a dramatic reduction of Pet precursor accumulation in cell lysates and mature Pet passenger in the media (Fig 5E). A similar reduction was also observed when the chimera was produced in the *E*. *coli* parent strain. Taken together, these results indicate that *E*. *coli* growth is not DegP-sensitive when Pet is produced with a pertactin signal sequence. Reduced accumulation of the chimera may permit viability.

### Loss of periplasmic proteins during *prn*-induced cell death

**Fig 6:**
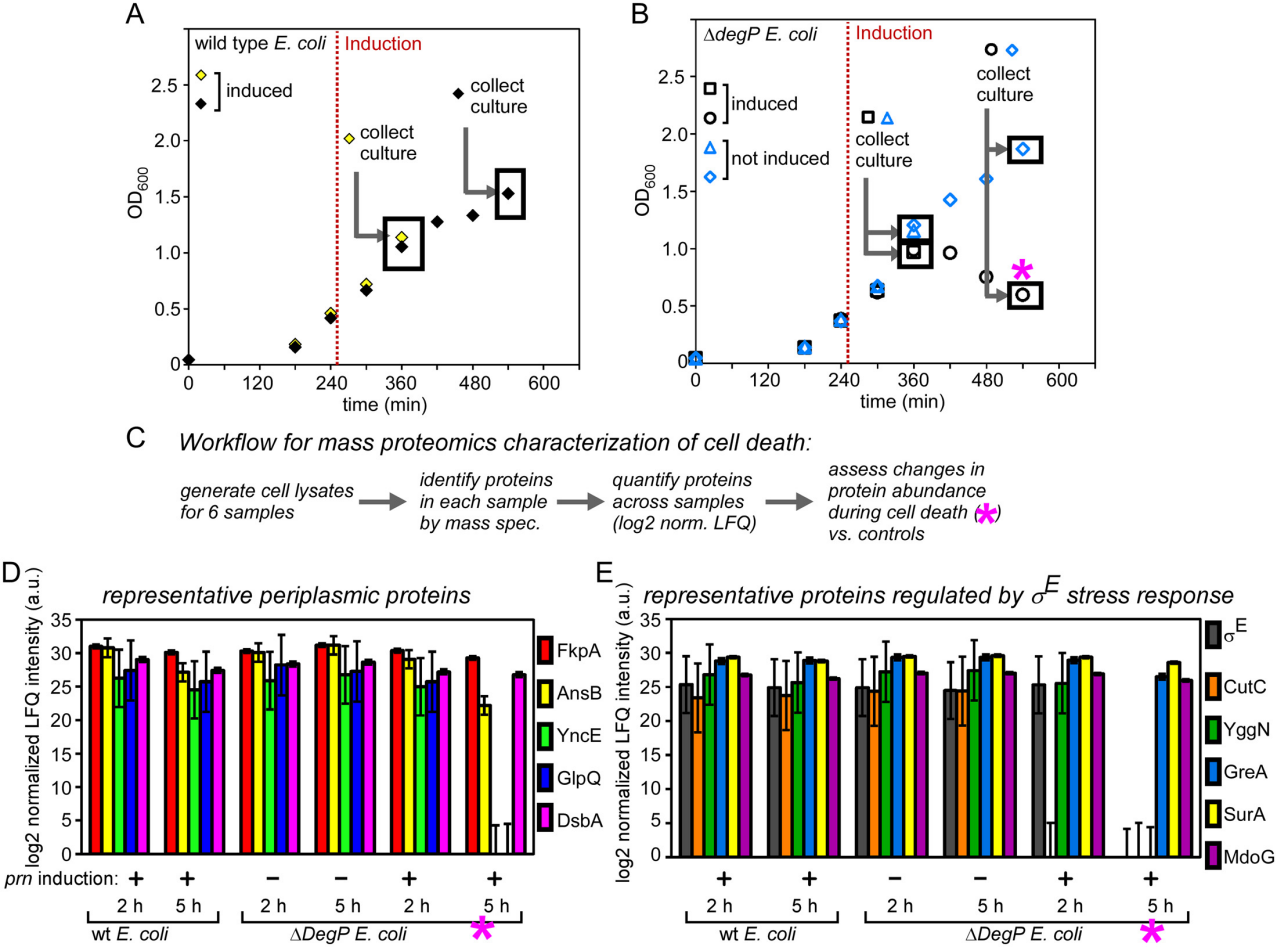
Analysis of proteome-wide changes during pertactin-induced death of *E*. *coli* Δ*degP*. (A, B) Expression of *prn* was induced in the presence (A) or absence (B) of a chromosomal copy of *degP* and cells were collected at 2 and 5 h post induction for mass spectrometry. For wild type *E*. *coli*, two samples were collected for mass spectrometry analysis (2 h and 5 h post *prn* induction). For the *degP* deletion strain, a total of four samples were collected (2 h and 5 h post *prn* induction and uninduced controls at the same time points). To express *prn*, construct *T5p-prn* was used and expression was induced in mid-log phase (see also Fig 2B). (C) Workflow to generate dataset for unbiased analysis of the proteome during *E*. *coli* death. (D) Quantification of representative periplasmic proteins across all experimental conditions indicates that overall periplasmic proteins decrease during death (last condition, see pink star in (B, C)). (E) Quantification of proteins in the σ^E^ stress response pathway reveals a loss of σ^E^ during death (last condition, pink star). Note that the σ^E^ protein itself along with CutC and YggN are undetectable in the last condition. Error bars correspond to the standard error (∑/mean) for each label-free measurement.

The results above demonstrate that in the absence of DegP, post-translational IM-translocation of pertactin is lethal. This finding was unexpected: There is no precedent in the literature for a link between DegP dependence, the IM translocation mechanism and cell death. To broadly explore the underlying cell death mechanism, we used quantitative mass spectrometry to measure changes in protein abundance across the proteome upon induction of this lethal phenotype. Defects in growth upon induction of *prn* in the *degP* deletion strain were first detectable 2 h post-induction, indicating that this represents an early stage of cell death (Fig 2B). At 5 h post induction, the OD_600_ had decreased significantly, indicating lethality. To capture both early and advanced stages of cell death, we analyzed changes to the proteome at both 2 h and 5 h after *prn* induction. Cells were grown as described previously (Fig 2B), and we collected samples for proteomic analysis at the chosen time points (Fig 6A and 6B). We compared *prn* induction in the *degP* deletion strain versus its parent strain and also included a control condition where *prn* expression was not induced in the *degP* deletion strain. Together, we collected samples in these three different conditions at 2 h and 5 h post induction, resulting in six samples overall (see Fig 6A and 6B for details).

Quantification of the entire proteome across our experimental conditions was performed using normalized label-free-quantification (LFQ), where measured peak areas from peptides specific to each protein were integrated, averaged and normalized [45,49]. Overall, 1897 proteins were quantitatively described across all mass spectrometry runs, representing approximately 50% of the total *E*. *coli* proteome (see Fig 6C and Methods for details).

Our results above indicated that lethality was caused by a periplasmic defect, motivating us to investigate changes to the periplasmic proteome during cell death. Of the most abundant periplasmic proteins across our experimental conditions, 82% (32 proteins) were ≥2-fold less abundant (or absent entirely) in the *degP* deletion strain at 5 h post *prn* induction. Representative examples are shown in Fig 6D. The striking reduction in the abundance of most periplasmic proteins suggests severe stress within the cell envelope. In contrast, we detected no loss of outer membrane porins (including OmpC, LamB and BamA) during *prn* expression in the absence of *degP*, and similarly stable levels for cytosolic markers including σ^70^ and DnaK (panel B in S2 Fig). These results are consistent with a model where expression of *prn* in the absence of *degP* disrupts protein homeostasis the periplasm.

### Collapse of the σ^E^ stress response during *prn*-induced cell death

The loss of periplasmic proteins as cells approached death led us to hypothesize that dysfunction of one or both of the two major periplasmic stress responses, σ^E^ and/or Cpx [73], might trigger lethality. We first investigated the σ^E^ stress response, in which the alternative sigma factor σ^E^ regulates expression of downstream genes [74–77]. Surprisingly, the σ^E^ protein itself was undetectable in the *degP* deletion strain 5 h after the induction of *prn*, but was detected in all other control conditions (Fig 6E). From this we conclude that the σ^E^ pathway is inactivated during death. Loss of σ^E^ could arise due to its degradation and/or reduced production. Alternatively, the σ^E^ protein could be sequestered at the IM where it becomes more difficult to detect using mass spectrometry. Indeed, when the σ^E^ pathway is inactivated the σ^E^ protein is sequestered at the IM [78]. Either of these scenarios is consistent with our data and would result in inactivation of the σ^E^ pathway. Since cytosolic σ^E^ is an essential component of *E*. *coli* [79], its absence is sufficient to explain the lethality of *prn* expression in the absence of DegP [80].

To test further for the activity of the σ^E^ response in our experimental conditions, we quantified 25 σ^E^-dependent gene products [74] detected in our dataset. We found that at least 13 σ^E^-dependent gene products are less abundant (≥2-fold) or undetectable in the *degP* deletion strain upon induction of *prn* (representative examples are shown in Fig 6E). These results indicate that collapse of the σ^E^ response contributes to lethality when pertactin accumulates in the periplasm in the absence of *degP*.

Next we investigated the Cpx pathway, which leads to *degP* upregulation upon cell envelope stress [81]. Although we identified 26 of the known Cpx-regulated proteins [82] in our proteomics dataset, most were of low abundance and therefore unsuitable for reliable quantification. Nevertheless, six Cpx substrates could be quantified to high reliability (panel C in S2 Fig). Although we observed some changes in these Cpx substrates during induction of *prn* (panel C in S2 Fig), these changes were less severe than the complete abrogation of σ^E^ and some of its substrates. Although we cannot rule out contributions from Cpx towards lethality, the observed changes in Cpx alone are insufficient to explain the lethal phenotype in *E*. *coli*. In contrast, the complete loss of σ^E^, which is essential, and loss of several σ^E^-dependent essential genes is sufficient to explain lethality.

## Discussion

It has previously been suggested that periplasmic chaperones participate in late steps of AT secretion, ensuring that the AT passenger remains in a high-energy, secretion competent conformation required for efficient transfer to BAM or TAM [25,31,53,83–86]. In particular, previous studies revealed a variety of effects that hint at an interplay between DegP and AT secretion, including increased *degP* expression upon AT expression [34,38,55], defects in cell growth and AT secretion in *degP* null strains [37,56,57] and binding of DegP to an AT passenger *in vitro* [37]. However, the results presented here represent the first identification of a lethal phenotype during AT expression in a *degP* deletion background. Moreover, to our knowledge this is the first observation of a link between the function of a periplasmic chaperone and the IM translocation mechanism of a secreted protein in Gram-negative bacteria

**Fig 7:**
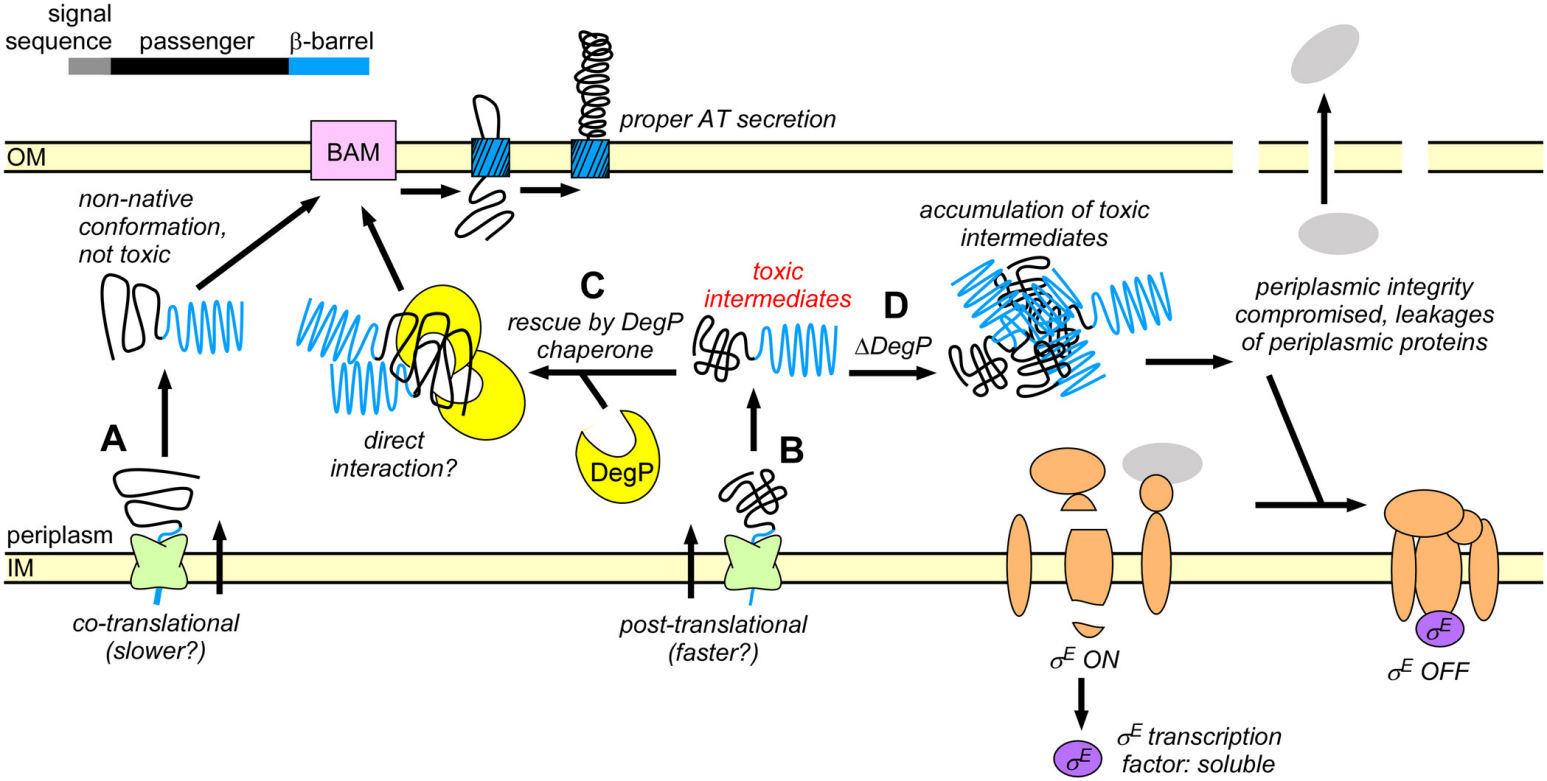
Model for the interplay between IM translocation, DegP chaperone activity, and the σ^E^ stress response. (A) Co-translational IM translocation results in passenger conformations in the periplasm that are tolerated in the absence of DegP. (B) Post-translational IM translocation results in conditional lethal AT conformations in the periplasm. (C) Lethality can be prevented by interactions with the chaperone DegP. (D) In the absence of DegP, accumulation of pertactin intermediates compromises periplasmic integrity and results in leakage of periplasmic material. This leads to inactivation of the σ^E^ stress response. σ^E^ inactivation is lethal and causes cell death.

The mechanistic connection between substrate IM import mechanism and DegP dependency may arise due to differences in IM translocation kinetics. Co-translational IM translocation is reported to be slower than classical post-translational IM translocation [5,8]. Hence changing the IM translocation rate could alter the conformation of the passenger as it enters the periplasm, akin to altered co-translational folding under different translation rates [87–89] (Fig 7). We propose a model where fast IM translocation via the PhoA signal sequence results in the rapid appearance of the substrate in the periplasm and subsequent accumulation of toxic conformations. These toxic conformations can be masked or avoided by DegP. Alternatively, reducing the rate of appearance of the passenger in the periplasm can avoid toxicity, for example during the slower, co-translational IM translocation of proteins bearing the DsbA signal sequence (Fig 7). This model predicts that the IM translocation mechanism and DegP work together to maintain *E*. *coli* secreted proteins in a non-native, non-toxic unfolded conformation.

Consistent with this model, we found that swapping the pertactin N-terminal signal sequence with the co-translational DsbA signal sequence was sufficient to restore *E*. *coli* growth in the absence of DegP, whereas the mainly post-translational PhoA signal sequence recapitulated the lethal phenotype. It is important to point out that both the DsbA and PhoA signal sequences are endogenous to *E*. *coli*. Hence while we cannot exclude that expressing the heterologous *B*. *pertussis prn* AT in *E*. *coli* may exacerbate lethality, the observation that these *E*. *coli* signal sequences were sufficient to recapitulate or reverse the phenotype demonstrates that the IM translocation mechanism is sufficient to alter the fate of a secreted protein in the *E*. *coli* periplasm.

Independent of the AT signal sequence, we found that properties of the AT passenger also contribute to lethality to some extent. Fusing the Pet signal sequence to the pertactin passenger only partially rescued the lethal phenotype, suggesting that the sequence and/or conformational properties of the AT passenger contribute to the viability phenotype (Fig 5). Although the pertactin signal sequence alone was not sufficient to render the Pet AT passenger lethal in the absence of DegP, these findings are ambiguous due to the dramatically reduced level of Pet accumulation (Fig 5E). Taken together, we found that ATs can avoid DegP dependence when at least one of two criteria are met: They are either translocated across the IM co-translationally, and/or have passenger folding properties capable of avoiding conformations that lead to lethality in the absence of DegP (Fig 7).

We characterized the lethality caused by pertactin synthesis in the *degP* deletion strain in an unbiased manner by measuring broad changes in the proteome. Although many periplasmic proteins were depleted during lethality in the absence of DegP (Fig 6D), we did not detect significant changes in the abundance of cytosolic or OM components (panel B in S2 Fig). Prior to pertactin production, we observed measurable levels of σ^E^ and σ^E^-induced proteins, which appears constitutive under our growth conditions. In contrast, we observed nearly complete inactivation of the σ^E^ stress response in the *degP* deletion strain upon *prn* induction. These results are consistent with lethality propagated from the periplasm, triggered by the periplasmic conformation of the pertactin passenger in the absence of DegP, which leads to collapse of the σ^E^ periplasmic stress response (Figs 6E and 7). Inactivation of σ^E^ could either be cause or consequence of leakage of soluble periplasmic material. The σ^E^ response is induced when DegS binds unfolded substrate proteins [75]. Therefore, its inactivation may start by release of DegS from its substrates, perhaps due to their dilution during periplasm leakage (Fig 7). In this scenario, unbound and inactive DegS prevents degradation of RseA, resulting in sequestration of the σ^E^ protein at the IM to RseA [75,80]. Membrane sequestration of the σ^E^ protein would also make it less likely to be detected by mass spectrometry, which could also lead to its absence in our proteomics assay. Consistent with this model, we also did not detect significant levels of other IM components of the σ^E^ machinery (DegS, RseA, RseP).

We were initially surprised that outer membrane proteins were largely unaffected by σ^E^ loss, since BamA and other OMPs are generally positively and negatively regulated by σ^E^ [74] and OMP levels overall were stable even after 5 h of *prn* expression. However, our results mirror those of Ades and coworkers, who found that an induced suppressor of σ^E^ function produced an analogous lethal phenotype with no apparent defects in outer membrane proteins [80]. Presumably, the unusually high stability of native OMPs [31,90] reduces their turnover rate, making them less susceptible to σ^E^ loss on our experimental time scale. Together, our unbiased characterization of changes across the proteome as cells approach death provides a unique view on time-resolved, global responses from the *E*. *coli* proteome when the periplasmic chaperone network collapses.

It is important to note that because the lethal DegP-dependent phenotype reported here may arise due to high-level production of a heterologous AT protein in *E*. *coli*, these results may have important implications for exploiting the AT secretion mechanism to produce heterologous proteins via autodisplay [91–93]. Furthermore, some ATs, including *pet*, are naturally encoded on plasmids. Differential reliance upon DegP could therefore prevent a Gram-negative pathogen from acquiring a plasmid via horizontal gene transfer. It will be important to investigate whether other Gram-negative species have a similar dependence upon DegP.

## Supporting Information

S1 Fig Primers used in this study. (TIF)

S2 Fig Quantification of marker proteins during proteomics experiments. (A) Quantification of DegP across all experimental conditions. (B) Quantification of representative marker proteins for the cytosol (σ^70^ and DnaK), the OM (LamB and BamA) and the OM-associated BamB. (C) Quantification of detectable proteins regulated by the Cpx stress response. No systematic decrease or increase of these Cpx-regulated proteins is observed during death (last condition, *prn* induction for 5 h in *E*. *coli DegP* deletion strain). Error bars correspond to the standard error (∑/mean) for each label-free measurement.
(TIF)

S3 Fig Deletion of *fkbA* and *skp* is not lethal during AT expression. (A) *E*. *coli* deletion strains from the BW30270 series [43] were transformed with plasmids that constitutively express *prn* or *pet*, as described in Fig 2A. Error bars in bold are standard deviations from at least three biological replicates. The vertical lines for *pet* transformed *degP*::*kan* indicates the spread of two biological replicates. (B) *prn* is expressed and the passenger is secreted in *fkpA*::*cm* and *skp*::*kan*. As a control the parent strain (BW30270) was used. Secretion is routinely assayed by the production of the processed passenger in whole cell lysate samples.
(TIF)
