# Supplementary figures and images for: DegP Chaperone Suppresses Toxic Inner Membrane Translocation Intermediates
Source: PLoS One. 2016 Sep 14;11(9):e0162922. doi: 10.1371/journal.pone.0162922 (PMC5023192; doi:10.1371/journal.pone.0162922)

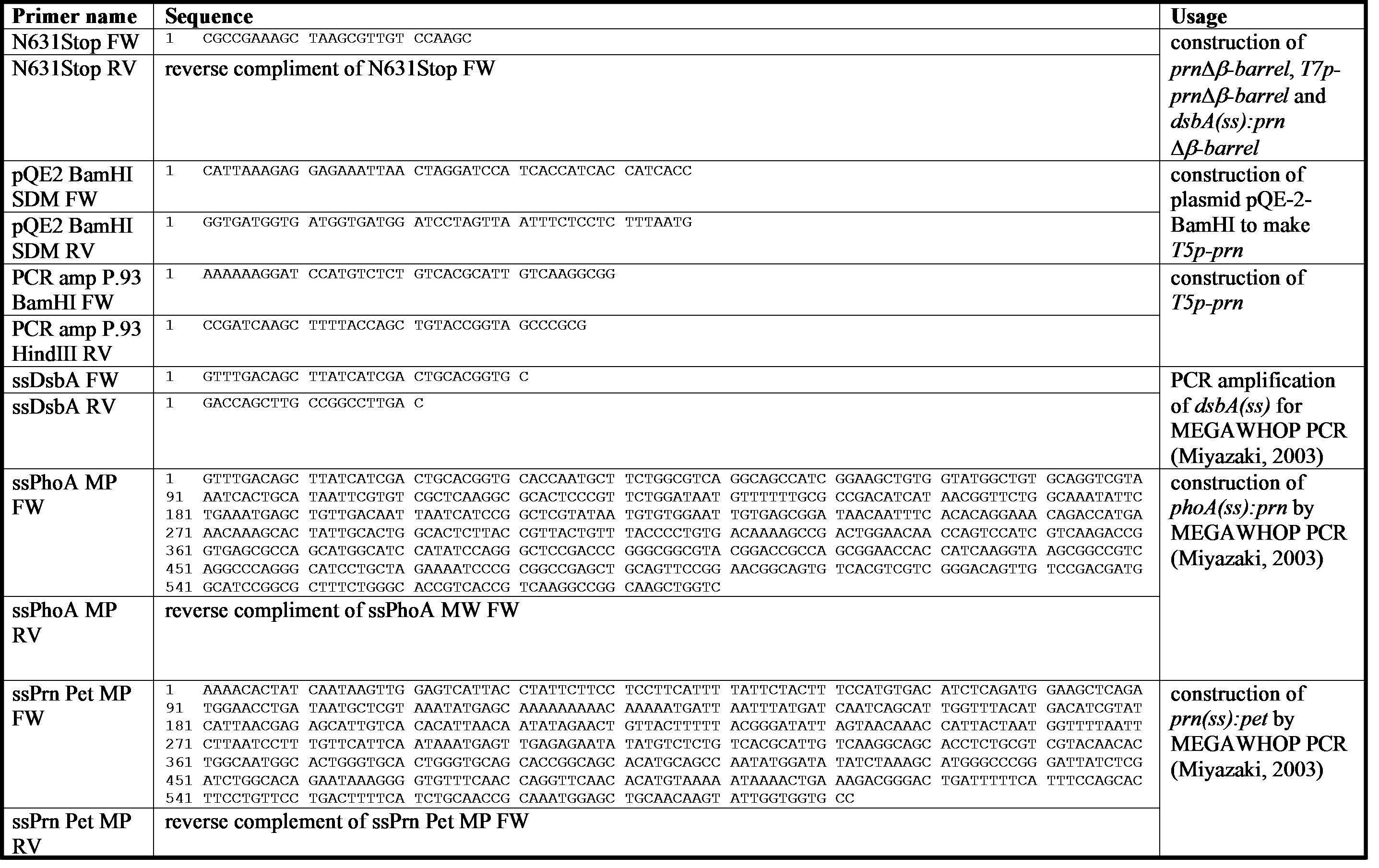

Supplement: S1 Fig — (TIF) [file pone.0162922.s001.tif]

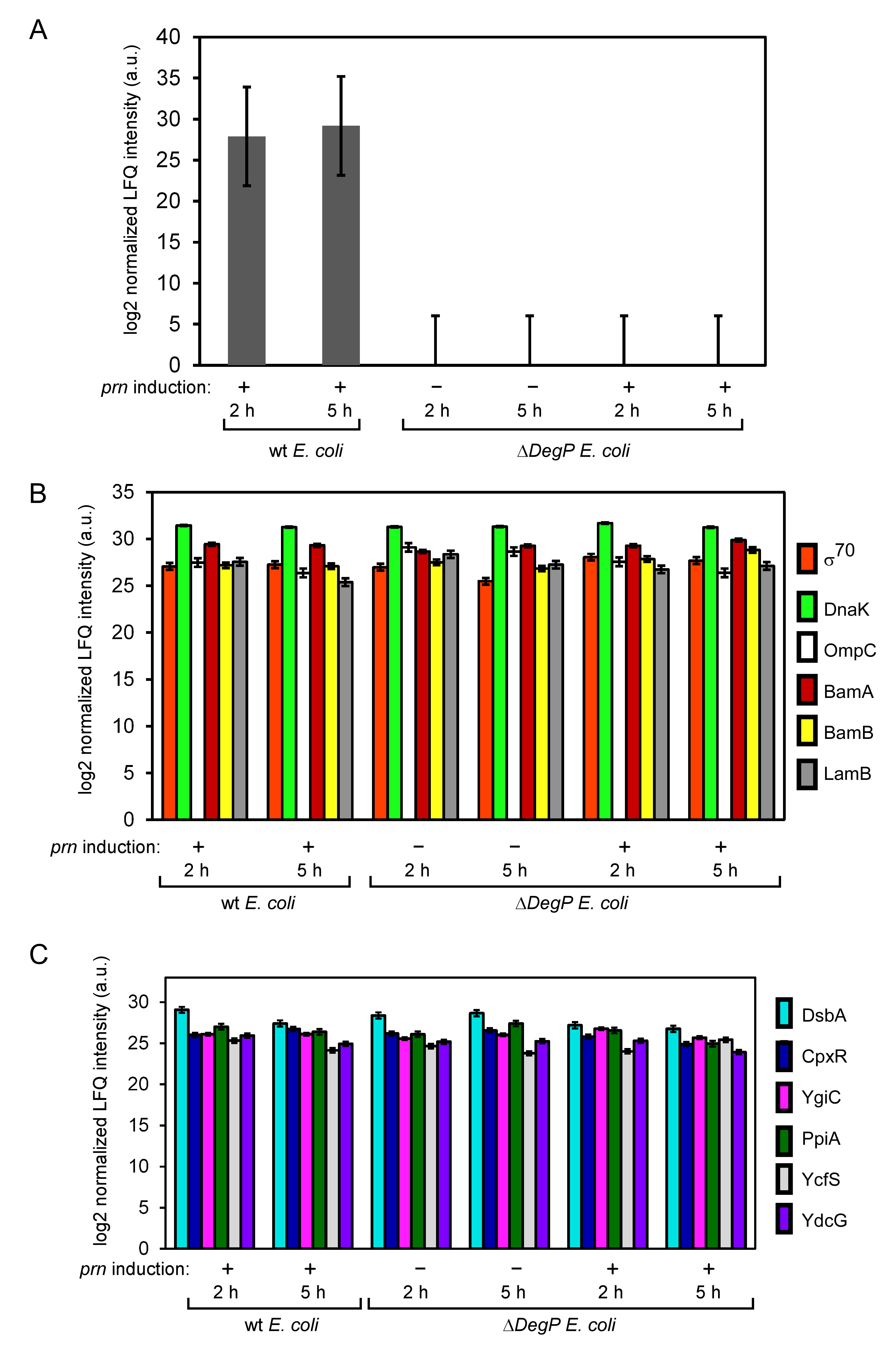

Supplement: S2 Fig — (A) Quantification of DegP across all experimental conditions. (B) Quantification of representative marker proteins for the cytosol (σ70 and DnaK), the OM (LamB and BamA) and the OM-associated BamB. (C) Quantification of detectable proteins regulated by the Cpx stress response. No systematic decrease or increase of these Cpx-regulated proteins is observed during death (last condition, prn induction for 5 h in E. coli DegP deletion strain). Error bars correspond to the standard error (∑/mean) for each label-free measurement. (TIF) [file pone.0162922.s002.tif]

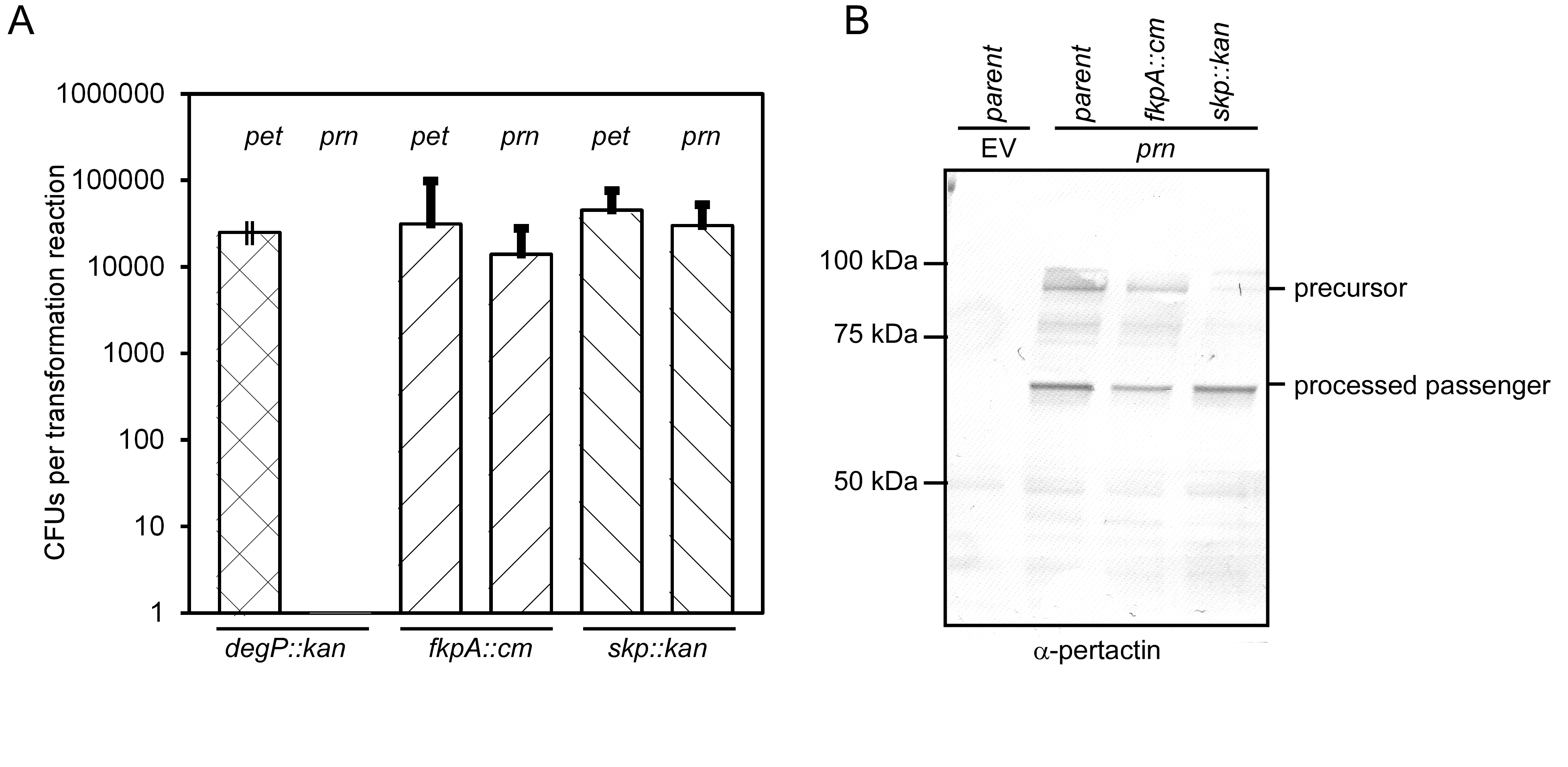

Supplement: S3 Fig — (A) E. coli deletion strains from the BW30270 series [43] were transformed with plasmids that constitutively express prn or pet, as described in Fig 2A. Error bars in bold are standard deviations from at least three biological replicates. The vertical lines for pet transformed degP::kan indicates the spread of two biological replicates. (B) prn is expressed and the passenger is secreted in fkpA::cm and skp::kan. As a control the parent strain (BW30270) was used. Secretion is routinely assayed by the production of the processed passenger in whole cell lysate samples. (TIF) [file pone.0162922.s003.tif]
